# Four attachment-based categories of emotion regulation in adolescent psychic troubles

**DOI:** 10.3389/fpsyg.2023.1133980

**Published:** 2023-05-19

**Authors:** Marion Robin, Luc Surjous, Jean Belbèze, Lucile Bonnardel, Claire Lamas, Jérôme Silva, Victoire Peres, Maurice Corcos

**Affiliations:** ^1^Department of Adolescent and Young Adult Psychiatry, Institut Mutualiste Montsouris, Paris, France; ^2^CESP, INSERM U1178, Paris-Saclay University, Villejuif, France; ^3^Paris Cité University, Paris, France

**Keywords:** emotion regulation, attachment, adolescents, empathy, facial affect recognition, alexithymia, defense mechanisms

## Abstract

**Introduction:**

Emotion regulation is altered in many psychiatric disorders in adolescence, but the understanding of mechanisms that underlie this alteration is still poor.

**Methods:**

The PERCEPT study explores alexithymia, empathy, facial emotion recognition (FER) and defence mechanisms in a sample of adolescents in psychiatric care (*n* = 61, 74% of girls, mean age = 15.03 y.o.), in relation with participants’ attachment styles.

**Results:**

Results revealed correlations between attachment dimensions and all of the emotion regulation variables, suggesting that attachment modalities have functional links with emotional regulation at its different levels: FER accuracy was inversely correlated with avoidant attachment, while affective empathy, difficulty in identifying feelings (alexithymia) and immature as well as neurotic defence mechanisms were positively correlated with anxious attachment. Moreover, attachment categories delineated distinct emotional perception profiles. In particular, preoccupied attachment included adolescents with the highest levels of facial emotion perception (sensitivity and accuracy) and of affective empathy, whereas detached attachment included adolescents with the lowest levels of these variables. Neurotic defence mechanisms and difficulty to identify feelings were correlated with preoccupied attachment; immature defence mechanisms and difficulty to describe feelings to others characterized fearful attachment.

**Discussion:**

These results suggest that attachment categories underlie emotion regulation processes in psychiatric disorders in adolescence. Theoretical and clinical implications are discussed.

## 1. Introduction

Emotion regulation is the ability of an individual to cope with an emotion or a set of emotions, to respond to an emotional experience. It is a complex psychological process that covers both the ability to trigger, inhibit, maintain or modulate one’s own affects in a broad sense, which includes physiological processes (e.g., heart rate), behaviors (e.g., laughter), cognitive processes and subjective feelings (Thompson, 2008; McRae and Gross, 2020). Into this multi-layered process, the perception of emotion, of others or within oneself, constitutes one of the early and central stages of emotional regulation across human emotional and interpersonal experiences (Hildebrandt et al., 2012; Addabbo et al., 2018). Facial emotion is one of the non-verbal components of communication, which provides information about the affective states of the subject who expresses it, but also about his or her behavioral intentions (Ekman et al., 1976; de Bonis et al., 1999). It also speaks from a social perspective, in which each emotion has a specific functionality. The perception (or recognition or identification) of emotions is therefore central to any form of communication in relationships. The concept of alexithymia describes the inability to detect its own emotions. It is a personality construct characterized by difficulty in identifying and describing feelings, a lack of fantasy, and a concrete and externally oriented thinking style (Sifneos, 1973c Taylor et al., 2003). Empathy, another dimension of emotion regulation, refers not only to emotion perception, but also to the ability to identify with or understand the perspective, experiences, or motivations of another individual and to comprehend and share his/her emotional state (Thompson et al., 2019). Finally, defence mechanisms are historically the first dimension of emotion regulation described (Freud, 1936/2001; Bailey and Pico, 2022). They were envisaged as a modality of reducing or removing any modification likely to endanger the integrity and constancy of the biopsychological individual (Laplanche and Pontalis, 1967).

In the last two decades, emotion regulation has increasingly been incorporated into models of psychopathology (Berenbaum et al., 2003; Mennin and Farach, 2007), and a growing number of data have pointed toward the existence of an impairment in a wide range of psychiatric disorders, visible in children, adolescents and adults, and impacting on family and peer relationships (Britton et al., 2010; Svaldi et al., 2012; Collin et al., 2013; Simcock et al., 2020). Most studies report emotional regulation deficits by describing a single dimension into a single diagnostic category, whether they explore alexithymia, facial emotion recognition (FER), defence mechanisms or empathy (Domes et al., 2009; Mitchell et al., 2014; Green et al., 2015; Jáni and Kašpárek, 2018) in adults and in adolescents. A main limit pointed out by these studies is the impossibility of concluding to a specificity of the described emotional particularity, as the comorbidities are so numerous, and as the described dimensions seem to cross nosographic categories (Domes et al., 2009; Preißler et al., 2010; Jovev et al., 2011; Mitchell et al., 2014; England-Mason, 2020). A number of studies concluded that difficulties in emotion perception were not linked to a particular diagnosis category, but, instead, appeared to be a transdiagnostic risk and/or maintenance factor (Svaldi et al., 2012; Cludius et al., 2020). Moreover, the few studies that have excluded most of comorbidities, for example in BPD, have also clearly limited the generalizability of their findings to the community of patients and deplored it (Minzenberg et al., 2006; Domes et al., 2008). Fewer articles described emotion perception abilities in adolescents, particularly in patients with severe psychiatric disorders, in a transnosographic perspective. Adolescents hospitalized in psychiatry are representative of adolescents with a severe emotional and social dysfunction, partly due to cumulative adversity (Robin et al., 2021). Delineating emotions perception profiles at this age could allow a better understanding of psychopathology and possibly orientate prevention and treatment (Kumari, 2020).

Impairment in empathy and FER were clearly described in anxiety, depression, and neurodevelopmental disorders (Mendlewicz et al., 2005; Habel et al., 2006; Chan et al., 2008; Kohler et al., 2010; Green et al., 2015; Graziano and Garcia, 2016; Tseng et al., 2017; England-Mason, 2020). However, results were more heterogeneous in the context of Eating Disorders (ED) and Borderline Personality Disorders (BPD). In both of these diagnoses, which are highly comorbid, FER and empathy are alternatively described as higher, comparable or lower than in healthy controls in adults and in adolescents (Domes et al., 2009; Robin et al., 2012; Mitchell et al., 2014; Cardi et al., 2015; Tchanturia et al., 2015; Dapelo et al., 2016). Renwick et al. (2015) explored this discrepancy among adults with Anorexia Nervosa, and observed that three separate clusters emerged when analyzing social-cognitive measures (emotional theory of mind), with high, medium, and low-level groups. Some explanation of the discrepancy in social cognition in these patients has been searched in the distinction of FER skills or empathy dimensions. This attempt revealed for example that BPD patients are highly responsive to the feelings of others (affective empathy), but are deficient in identifying/describing feelings and in taking the perspective of others (cognitive empathy) (New et al., 2012; Dinsdale and Crespi, 2013).

Attachment modalities toward caregivers have been shown to influence emotion regulation (Fonagy and Luyten, 2009; Timpano and Port, 2021). Attachment orientations have important implications for emotion regulation and health, and attachment insecurity is associated with deficits in neural structure associated with emotion regulation (Mikulincer and Shaver, 2019). Although attachment disorders and emotional regulation have both been explored in a variety of diagnostic areas, their relationship has been most extensively studied in BPD. Indeed, patients with this diagnosis show significant emotional dysregulation (this is a diagnostic criterion) and in this disorder, attachment is particularly insecure (Miljkovitch et al., 2018). According to Bowlby’s (1969, 1982) concept of the internal working model, individuals develop models of self as more or less lovable and of others as more or less reliable and loving, depending on the quality of received care. In this line, both dimensional and categorical measures of attachment were built in a complementary way. Dimensional measures of attachment focus on attachment anxiety (negative model of the self, preoccupation with the availability and responsiveness of others with expectation of abandonment) and avoidance (negative model of others and devaluation of the importance of close relationships) (Main, 1995; Hazan and Shaver, 1987; Brennan et al., 1998). Bartholomew (1990) defined four prototypic attachment categories based on these underlying dimensions: secure (positive self and other model), fearful (negative self and other model), preoccupied (negative self model, positive other model) and dismissing (negative other model, positive self model). Thus, the negative model of self (fearful and preoccupied styles) relates to anxiety, while the negative model of others relates to avoidance (secure and dismissing styles). Attachment is considered disorganized when no category emerges in a stable manner. These two descriptive modalities of attachment (dimensions and categories) are used alternately according to the studies, which extends their interest but also makes comparisons difficult from one study to another, depending on whether the method is dimensional or categorical (Ravitz et al., 2010).

Studies have highlighted that BPD patients mostly show preoccupied, fearful and disorganized attachment patterns (Levy et al., 2005; Fonagy and Luyten, 2016), both of which have been hypothesized to influence emotion regulation in BPD (Skodol et al., 2002). Individuals with high levels of attachment anxiety, because of their hypersensitivity to rejection and abandonment, are supposed to develop hypersensitivity to external features in others, including affective facial expressions (Mikulincer and Shaver, 2007, 2008). Individuals high on attachment avoidance, by contrast, are often particularly poor at “reading” facial expression in others because of their tendency to deactivate attachment concerns (Van Heel et al., 2019). Studies on attachment modalities and FER are less frequent in other psychiatric diagnoses, but have also identified an influence of attachment modalities on emotion perception in Anorexia Nervosa (Doba and Nandrino, 2020). Studies on this topic are still lacking in other clinical areas and the understanding of the links between attachment and emotional regulation remains very partial. A review of the links between attachment and empathy, however, suggested a significant link between empathy and avoidant and secure attachment, but an insignificant link between empathy and anxious attachment (Xu et al., 2022). As for the links between alexithymia and attachment, several studies have shown negative correlations between alexithymia and attachment security (Troisi et al., 2001; Montebarocci et al., 2004; Wearden et al., 2005). This is consistent with Bowlby's (1969) view that secure attachment is fundamental to allowing exploration of internal emotional states.

In the field of emotional regulation, defence mechanisms have been less explored than other aspects of emotion regulation in the last years. They constitute a modality of reducing or removing any modification likely to endanger the integrity and constancy of the biopsychological individual (Laplanche and Pontalis, 1967). They allow protecting the personality of the subject by decreasing the anguish, which can be felt in a given situation, thus they are a central element of the emotional regulation. Recent studies have demonstrated that defences can be arranged hierarchically based on their usual level of adaptiveness, and that defensive functioning tends to improve over the course of psychotherapy in stepwise fashion (Drapeau et al., 2003; Bond and Perry, 2004; Perry and Bond, 2012). Various authors have attempted to describe them precisely according to psychiatric diagnoses, most often using the Defence Style Questionnaire (Perry and Cooper, 1986; Bond, 1992; Paris et al., 1996; Koenigsberg et al., 2001), but their transnosographic role in emotion regulation and perception has still to be investigated. In this line, Ciocca et al. (2020) have recently demonstrated that attachment styles – preoccupied and fearful, and defence mechanisms – immature and neurotic, had a substantial impact on psychological distress in a sample of students.

In sum, there are a number of arguments to suggest that there is a link between attachment modalities and emotional regulation, a link that seems to underlie psychiatric diagnoses (Wearden et al., 2005; Mikulincer and Shaver, 2007, 2008). This link has so far been explored mainly separately for each dimension of emotional regulation (Ciocca et al., 2020; Xu et al., 2022), whereas we believe that a common analysis could be useful in order to understand psychiatric psychopathology in a more unified way. Similarly, research on attachment finds that the dimensions and categories make different but complementary contributions, which encourages their analysis in a single study (Bartholomew and Horowitz, 1991; Ravitz et al., 2010). Finally, these links between emotional regulation and attachment have not been explored in adolescents independently of their psychiatric diagnoses. For all these reasons, the aim of the present work is to explore, in an adolescent psychiatric sample, the links of attachment dimensions and categories with emotion regulation in a transnosographic perspective. Our first hypothesis is that the dimensions of attachment are correlated with all levels of emotional regulation explored: emotional recognition, empathy, alexithymia, defence mechanisms. Our second hypothesis is that it is possible to distinguish different modalities of emotional regulation according to the categories of attachment in these patients.

## 2. Materials and methods

### 2.1. Participants

The PERCEPT study included 61 inpatient adolescents admitted in an urban psychiatric crisis unit during 6 months, from October 2018 to April 2019 (Institut Mutualiste Montsouris, Paris, France). The unit receives adolescents referred by psychiatrists for any psychiatric crisis situation. The study was proposed to 65 patients successively hospitalized, in order to evaluate their emotion regulation with regard to their attachment styles. The inclusion criteria were all hospitalized patients who agreed to the research (as well as their parents); and the exclusion criteria were the impossibility for a patient to participate in an experimental test, either for a reason of refusal or for an incapacity related to their condition (incompatible state of agitation or incapacity to understand the task). The average length of hospitalization for these patients was 22 days and recruitment was done at the end of the hospitalization. The questionnaires were given to the patient during the last week of hospitalization, and they returned them to the investigator on the day of the experiment. Four patients refused to participate and one patient was not clinically able to. Procedures were approved by the Ethics Committee of Necker Hospital, Paris, France (CPP-IDFII-2013-09-10).

All participants were native European adolescents, including 74% of girls (sex assigned at birth), aged from 13 to 19 years old (*m* = 15.03 y.o., sd = 1.3). Most of them (52.9%) belonged to families with high economic status. Fifty-seven patients (93.4%) were currently under psychotropic medication, and fifty patients (81.9%) had received at least one psychotropic treatment prior to hospitalization. Twenty-seven patients (44.3%) had experienced self-injury, and thirteen patients (21.3%) had shown aggressive behaviors. Regarding patients’ medical profiles, the variety of diagnoses has led us to group them into categories: 36.1% of patients were diagnosed with an anxiety or depressive disorder, 39.3% with a BPD, 32.8% with Eating Disorders, and 22.9% with a psychosis (schizophrenia, autism, or pervasive developmental disorder). Most patients had more than one diagnoses (2.2 diagnoses on average per patient), and in this case, the main diagnosis that was retained was the most clinically invasive. Distribution of these diagnoses according to the four categories of attachment was measured for secure attachment (33% anxiety-depressive patients, 22% BPD patients, 22% of patients with ED, and 22% of patients with psychosis), fearful attachment (27% anx-dep, 33% BPD, 33% ED, 7% psychosis), preoccupied attachment (21% anx-dep, 33% BPD, 33% ED, 12% psychosis), and detached attachment (23% anx-dep, 23% BPD, 15% ED, 39% psychosis).

### 2.2. Procedure

All subjects completed a research protocol, including a self-reported questionnaire assessing socio-demographic, psychopathological data, and the behavioral task. Diagnoses of axis-I and axis-II disorders according to the DSM 5 criteria were collected from the patient’s records at the end of the hospitalization.

Alexithymia was assessed with the 20-item Toronto Alexithymia Scale (TAS-20; Bagby et al., 1994a,b), a 5 points Likert-scale questionnaire. The 20 items of the TAS are clustered into three factors corresponding to the theoretical dimensions of alexithymia: Difficulty Identifying Feelings (DIF), Difficulty Describing Feelings (DDF), and Externally Oriented Thinking (EOT). TAS-20 scores are reliable, and the three-factor structure is replicable (Bagby et al., 1994a). The TAS-20 is currently the most widely used measure of alexithymia, and considerable work has gone into testing its reliability and validity (Bagby et al., 1994b; Parker et al., 2003; Taylor et al., 2003).

For the self-reported assessment of empathic abilities, the Interpersonal Reactivity Index (IRI; Davis, 1983) was used. Participants had to rate 32 statements describing their empathic abilities with scores ranging from 0 (that does not describe me well) to 4 (that describes me very well). The questionnaire consists of two dimensions of affective and cognitive empathy, and has good reliability with a Cronbach’s alpha of 0.78.

Defence mechanisms were evaluated with the Defence Style Questionnaire (DSQ) (Bond and Vaillant, 1986), which is a 9-point Likert-scale that measures the conscious derivatives of different defence mechanisms through 88 items. The defence score is the average response of items contributing to the specific set. A higher score indicates greater use of the respective defence mechanism. According to Andrews et al. (1989), a factor analysis has identified 25 separate defences that cluster into three higher order defence styles by their functional adequacy: mature, including suppression, task orientation, anticipation, sublimation, and humour; neurotic, including reaction formation, undoing, inhibition, withdrawal, idealization, and pseudo-altruism; and immature, including projection, passive aggression, acting out, omnipotence/devaluation, help rejecting, fantasy, isolation, splitting, projective identification, regression, somatization, denial, affiliation, and consumption. The DSQ has been shown to discriminate control populations from psychiatric outpatients, as well as from patients with eating disorders (Steiger et al., 1989) and anxiety disorders (Bond and Vaillant, 1986; Andrews et al., 1989; Martin et al., 2019).

In order to assess attachment categories, we used the Relationship Questionnaire (RQ, Bartholomew and Horowitz, 1991), which consists of four paragraphs giving rise to both categorical and continuous data. The categorical measure is a forced-choice measure whereby participants select one of the four paragraphs indicating their attachment style as secure, preoccupied (anxious), fearful (anxious and avoidant), or dismissive (avoidant). The continuous measure, in which individuals indicate the extent to which they resemble each of the four attachment styles on a 7-point scale (1 = not like me at all to 7 = very much like me), gives rise to four continuous scales. The RQ has demonstrated adequate predictive validity and test–retest reliability, and is a widely used measure of attachment modalities (Scharfe and Bartholomew, 1994; Herzberg et al., 1999; Wongpakaran et al., 2021).

All these tools are frequently used in adolescent research samples.

### 2.3. Emotion recognition

Emotion recognition was assessed with a task that comprised 36 trials presented in a random order. The stimuli were taken from the empirically valid and reliable pictures of the facial affect series of Ekman and Friesen (1976). Each trial began with a neutral face gradually morphed into one of the six prototypical emotions—sadness, anger, happiness, disgust, surprise, and fear—according to forty 2.5% incremental stages. Each picture was presented for 500 ms followed immediately by the next morphed face in the sequence. Each trial therefore consisted of a 20 s continuum. We used the pictures of three men and three women, who were each expressing the six emotions of interest. Each emotion was thus presented six times. Participants were asked to report the emotion expressed whenever they thought they had identified it, by clicking on one of the six corresponding boxes. They were also told that they could change their initial response at any time and as often as necessary by clicking again, and that they had to indicate their final choice at the end of each trial (40th stage). With this paradigm, two FER skills were scored: accuracy was measured by the final success rate (i.e., percentage of correct response at 100% expression), and sensitivity by the precociousness of a right emotional recognition. It is measured by the difference between the final stage (40th stage) and the mean number of stages required for the accurate identification of facial emotions, across the trials in which the participants successfully recognized the final expression (Sensitivity = 40 − n). The greater is the difference between 40 and the number of images needed, the earlier the emotional recognition and the higher the sensitivity. The first variable describes the ability to recognize emotion independently of time, while the second describes its rapidity.

### 2.4. Data analysis

Emotional variables were first compared to age, sex and psychotropic number and using spearman correlations and student *t*-tests. Spearman correlations were also performed between emotional variables and attachment dimensions in the whole sample. We then used Kruskal-Wallis tests to determine statistically significant differences between mean levels of empathy, FER, Alexithymia and Defence mechanisms in the four attachment categories, as a one-way ANOVA non-parametric equivalence (R software).

## 3. Results

The effects of age, sex and medication on emotion variables are reported in Table 1. We observed significant positive effect of age on FER sensitivity, of gender on affective empathy and defence mechanisms: affective empathy, as well as immature and neurotic defences mechanisms were more frequent in young women than in young men.

**Table 1:** Correlations between age, psychotropic number and emotion regulation variables, and *t*-tests of emotion regulation variables by sex: facial emotion recognition sensitivity and accuracy, affective empathy, cognitive empathy, alexithymia, mature, immature and nevrotic defence mechanisms (Aff_emp, Affective empathy; Cogn_emp, Cognitive empathy; DM, Defence Mechanisms; Nb, number).

*n* = 61	Sensitivity	Accuracy	Aff_emp	Cogn_emp	Alexithymia	Mature DM	Imm DM	Nevr DM
Age	0.38**	0.14	0.08	0.18	−0.26	0.04	−0.12	−0.08
Psychotropic Nb	−0.16	0.2	−0.06	0.21	0.02	−0.1	−0.14	0.06
Sex								
Male (*n* = 16)	31.7 (±4.2)	0.76 (±0.1)	27.9 (±8.1)	25.5 (±6.9)	61.5 (±11.5)	38.8 (±14.1)	65.9 (±20.9)	30.4 (±13.0)
Female (*n* = 45)	29.0 (±3.8)	0.79 (±0.1)	35.4 (±8.8)	28.8 (±7.4)	59.7 (±12.7)	41.6 (±11.7)	87.2 (±23.4)	40.4 (±12.8)
	*t* = 418	*t* = 248	*t* = 97.0**	*t* = 148	*t* = 192	*t* = 160	*t* = 96.5**	*t* = 110*

*p < 0.05; **p < 0.01.

### 3.1. Emotion regulation and attachment dimensions

Correlations between emotion regulation variables are reported in Figure 2:

- Anxious attachment was positively and significantly correlated to affective empathy, Nevrotic and Immature Defence mechanisms and Difficulty in Identifying Feelings.
- Avoidant attachment was negatively and significantly correlated to FER Accuracy.

**Figure 1:**
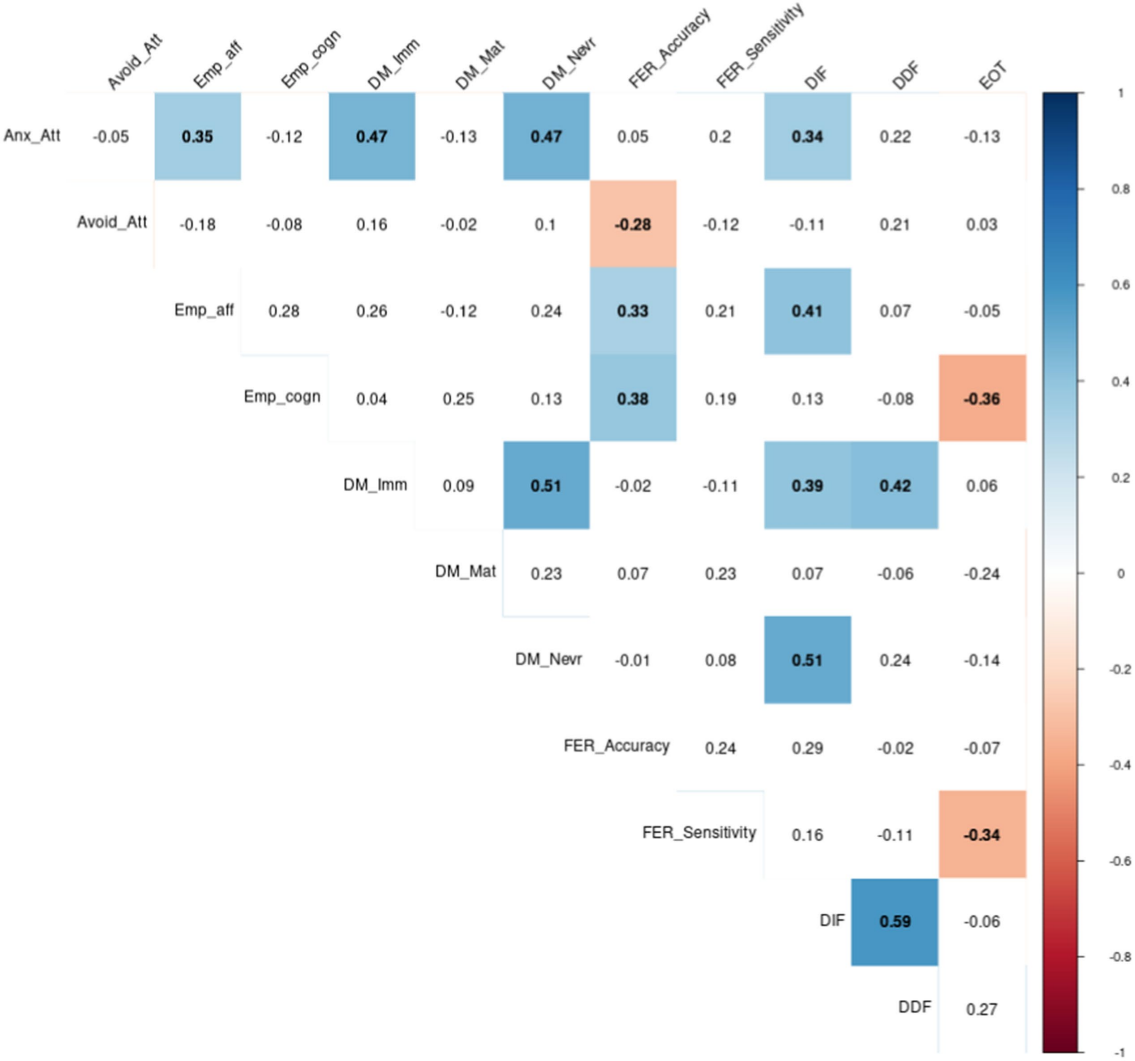
Correlations between emotion regulation variables: Attachment, Empathy, Defence Mechanisms, Facial Emotion Recognition and Alexithymia (Anx_att, Anxious attachment; Avoid_att, Avoidant attachment; Emp_aff, Affective Empathy; Emp_cogn, Cognitive Empathy; DM_Imm, Immature Defence Mechanism; DM_Mat, Mature Defence Mechanism; DM_Nevr, Nevrotic Defence Mechanism; FER_Accuracy, Facial Emotion Recognition Accuracy; FER_sensitivity, Facial Emotion Recognition Sensitivity; DIF, Difficulty Identifying Feelings; DDF, Difficulty Describing Feelings; EOT, External Oriented Thinking).*p* < 0.05.

We also observed correlations between emotional regulation variables. Among them:

- Empathy scores were positively correlated with DIF (affective empathy) and FER accuracy (affective and cognitive empathy), but negatively correlated with EOT (cognitive empathy).
- FER sensitivity was negatively correlated with EOT (alexithymia).
- Alexithymia was positively correlated with defence mechanisms: DIF score was correlated to Immature and Nevrotic defence mechanisms, while DDF score was correlated to Immature dimension. DIF score was also positively correlated with affective empathy, and EOT was negatively correlated with cognitive empathy.

### 3.2. Emotion regulation and attachment categories

Distribution of Empathy, Facial Emotion Recognition, Alexithymia and Defence Mechanisms levels by attachment categories and Kruskal-Wallis tests are reported in Table 2. It revealed contrasts, mostly for affective empathy and FER sensitivity among attachment categories, which, respectively, reached and tended to reach significance. Their highest scores were both observed in the preoccupied attachment category. It also revealed significant discrepancies in alexithymia (DIF and DDF) and defence mechanisms (total, neurotic and immature) among attachment categories. Considering the cut-off score of alexithymia, only preoccupied and fearful patients were alexithymic. The highest scores of DIF and DDF were observed in fearful attachment, as was the highest score of immature defence mechanisms, whereas highest scores of total and neurotic defence mechanisms were observed in the preoccupied attachment style.

**Table 2:** Means of empathy, facial emotion recognition, alexithymia and defence mechanisms levels by attachment categories and Kruskal-Wallis tests results (Att, attachment; Emp_aff, Affective empathy; Emp_cogn, Cognitive empathy; FER, Facial Emotion Recognition; TAS, Toronto Alexithymia Scale; RQ, Relationship Questionnaire).

Att category (RQ)	Secure att (27%)	Preoccupied att (31%)	Fearful att (20%)	Dismissing att (22%)	Test
Emotions					
Empathy					
Total	58.4 (±11.6)	67.6 (±12.3)	62.5 (±14.5)	57.2 (±14.8)	*t* = 3.8; *p* = 0.29
Aff_emp	31.2 (±7.97)	38.9 (±7.78)	33.8 (±11.6)	29.3 (±6.48)	***t* = 8.4; *p* = 0.04**
Cogn_emp	27.1 (±6.60)	28.7 (±7.29)	28.7 (±7.85)	27.9 (±9.14)	*t* = 0.58; *p* = 0.9
FER					
Sensitivity	11.2 (±2.2)	12.0 (±3.7)	8.6 (±4.2)	8.1 (±5.4)	*t* = 6.6; *p* = 0.09
Accuracy	0.81 (±0.1)	0.81 (±0.1)	0.76 (±0.1)	0.76 (±0.1)	*t* = 4.8; *p* = 0.19
Alexithymia					
TAS tot	54.5 (±14.3)	61.7 (±10.4)	65.7 (±10.6)	59.7 (±12.7)	*t* = 5.6; *p* = 0.12
TAS 1	19.4 (±6.4)	25.6 (±5.8)	26.1 (±5.5)	19.3 (±4.8)	***t* = 13.2; *p* < 0.01**
TAS 2	13.9 (±5.0)	16.5 (±4.8)	20.0 (±4.7)	17.6 (±4.6)	***t* = 8.5; *p* = 0.04**
TAS 3	21.2 (±5.8)	19.6 (±4.1)	19.6 (±4.4)	22.8 (±7.6)	*t* = 1.95; *p* = 0.6
Defence mechanisms					
Sum	133 (±32.5)	183 (±30.8)	174 (±39.4)	157 (±24.0)	***t* = 15.6; *p* < 0.01**
Nevrotic	26.6 (±10.4)	48.9 (±9.0)	41.1 (±11.3)	34.9 (±10.3)	***t* = 24.4; *p* < 0.001**
Immature	67.0 (19.8)	92.6 (21.4)	94.3 (29.2)	77.6 (16.3)	***t* = 12; *p* < 0.001**
Mature	39.4 (±12.0)	41.8 (±12.6)	39.1 (±11.8)	44.3 (±12.9)	*t* = 0.8; *p* = 0.84

*p* < 0.05.

## 4. Discussion

Adolescents hospitalized in psychiatry are characterized by severe relational dysfunction, but so far little is known about the way these patients process emotion regulation.

### 4.1. Attachment modalities and emotion regulation

Our results revealed correlations between attachment dimensions and all of the emotion regulation variables: facial emotion recognition, empathy, alexithymia and defence mechanisms. While FER accuracy was correlated to avoidant dimension, the other three dimensions were correlated with the anxiety dimension of attachment. The fact that correlations were found on all the dimensions of emotional regulation explored suggests that attachment modalities have functional links with emotional regulation at its different levels.

The results also revealed that attachment categories might delineate distinct profiles of emotion regulation in this population sample. In particular:

- secure patients had the lowest scores of alexithymia and defence mechanisms, and intermediate levels of empathy and FER skills.
- preoccupied attachment included adolescents with high levels of alexithymia, FER skills (sensitivity and accuracy), affective empathy and defence mechanisms.
- dismissing patients showed low levels of alexithymia (DIF and DDF), FER skills, affective empathy and defence mechanisms.
- fearful attachment included adolescents with high levels of alexithymia and defence mechanisms, but medium or low levels of FER skills and empathic concern.

The distribution of psychiatric diagnoses according to the four categories of attachment revealed that anxiety-depressive patients were predominantly represented by secure attachment, while BPD and ED patients were mostly included in preoccupied and fearful categories. Psychotic troubles were more represented in detached attachment.

#### 4.1.1. Attachment dimensions and categories with FER

The accuracy variable in FER was correlated with the avoidant dimension of attachment, in the sense that the more avoidant the subject’s attachment, the less able he or she was to identify emotions on another’s face. This study is in line with previous research on attachment dimensions and emotion recognition in BPD patients, which described individuals with BPD and healthy controls that score high on attachment avoidance showing impaired recognition of facial expressions in a RMET study (Van Heel et al., 2019). Authors commented that this result was consistent with findings that individuals with high avoidant attachment are often poor at recognizing facial emotions because they tend to deactivate attachment needs and concerns (Fonagy et al., 2003). However, other studies have shown different results. In previous studies, individuals with high levels of attachment anxiety had been described as developing hypersensitivity to affective facial expressions of others (Niedenthal et al., 2002; Mikulincer and Shaver, 2007, 2008). There is currently no consensus as to whether attachment phenomena are inherently categorical or dimensional (Ravitz et al., 2010), and it is perhaps the dual consideration of attachment via dimensions and categories at the same time that allows us to see the extent to which the two anxious and avoidant dimensions combine their effects with respect to facial emotion recognition. Statistical analyses suggested that attachment categories allowed a distinction of emotional profiles, although, probably due to the sample size, these results did not reach statistical significance for FER accuracy. Subjects with low-avoidant attachment had significantly better FER abilities (significant dimensional correlations), but between the two low-avoidant categories (secure and preoccupied), FER scores would be highest in the preoccupied style (tendency in categorical analyses). This suggests that the subjects with the best perception abilities have an insecure attachment, and it is consistent with the observations of Niedenthal et al. (2002) that insecure individuals showed better FER abilities than healthy subjects. Furthermore, it is those who meet both the condition of having a negative self-model and a positive other-model who would be most likely to observe others with acuity. This suggests that this is the relational situation that involves the most dependence and high motivation to seek closeness with a person deemed more reliable than oneself, which could lead one to look more intensely for signs of its availability to regulate one’s own emotions.

#### 4.1.2. Attachment dimensions and categories with empathy

Our results revealed a positive correlation between anxious attachment and affective empathy. The highest level of affective empathy was also observed in the preoccupied style beyond the categorical analyses. These results reinforce the hypothesis that they are links between both attachment and empathy, even if the nature of these links is still unclear today. A recent meta-analysis of Xu et al. (2022) described a correlation between cognitive empathy and secure attachment in children and adolescents, and a low significant negative correlation between empathy and avoidant attachment. There is some evidence to support a link between empathy and attachment, but results vary widely depending on contextual, developmental, and methodological factors (Cassidy and Stern, 2018). Our results are not in line with the previous results, which, for the most part, are not based on clinical populations (Xu et al., 2022). Neurodevelopmental studies found that the development of affective empathy was related with that of the limbic amygdala, whereas the development of cognitive empathy was consistent with that of the prefrontal cortex, a brain region related to higher and more mature cognitive functions (Cui et al., 2008). Our results are consistent with the notion that in some psychological disorders such as BPD, amygdala hyperactivity develops in parallel with prefrontal disconnexion (New et al., 2007), and this could be associated with the excessive development of affective empathy unbalanced by cognitive empathy.

The predominant repartition of BPD in negative self-model attachment categories is in significant agreement with the literature, as many authors have described that adolescent or adult BPD patients show preoccupied or fearful attachment (Deborde et al., 2012). According to Fonagy and Bateman (2006), both insecure attachment and dysfunctional affect regulation constitute predisposing factors for the development of BPD. In our sample, negative self-model attachment categories (preoccupied and fearful), in which BPD were mostly represented, included high level of anxious attachment, alexithymia and defence mechanisms. While research consistently showed that BPD patients have biases in mental state attribution (e.g., evaluate others as malevolent) and are alexithymic, research focusing on emotion perception and empathy is less consistent (Preißler et al., 2010; Roepke et al., 2013; Kiliç et al., 2020; Németh et al., 2020). The empathy paradox refers to empirical evidence that BPD subjects may demonstrate enhanced empathy in spite of impaired interpersonal functioning (Krohn, 1974; Dinsdale and Crespi, 2013). A review of 28 studies on empathy in BPD reported comparable levels of evidence for enhanced, preserved, and reduced empathic skills in individuals with BPD (Dinsdale and Crespi, 2013). Similarly, studies have shown heterogeneous theory of mind abilities in ED (Renwick et al., 2015), with clusters of patients, which were not differing in ED symptoms, comorbidity features or treatment characteristics. In our study, the predominant repartition of AN and BPD in preoccupied and fearful categories of attachment suggests that the unexplained heterogeneity in social cognitions in some studies may be partly attributable to attachment heterogeneity, in AN as in BPD, which represents more than a high comorbidity, a very intricate common functioning (Khosravi, 2020). It suggests that heterogeneity in attachment in BPD and ED profiles may contribute to this discrepancy in literature data, with patients associating high empathic skills as a result of high anxious and low avoidant attachment, and patients with lower empathic skills as a result of lower anxious and higher avoidant attachment than in preoccupied category.

#### 4.1.3. Attachment dimensions and categories with alexithymia

Correlations between attachment dimensions and alexithymia total scores and subscales revealed that the difficulty in identifying feelings was linked with anxious dimension. Alexithymia, a personality construct characterized by this difficulty to identify and describe feelings, a lack of fantasy, and a concrete and externally oriented thinking style (Sifneos, 1973; Taylor et al., 2003), is largely described among psychiatric disorders in adults and adolescents (Bankier et al., 2001; Deborde et al., 2012; Leweke et al., 2012).

Among attachment categories, comparison of alexithymia total scores revealed that the difficulty in identifying feelings was mostly present in patients with anxious attachment (preoccupied and fearful), whereas the difficulty in describing feelings was more specifically described in fearful category of attachment. Our results echo previous studies describing that in young men with depressive symptoms, those with preoccupied or fearful categories had a higher prevalence of alexithymia than those with a dismissing category (Troisi et al., 2001). Wearden et al. (2005) also described association between negative model of the self, negative affectivity and fearful/preoccupied attachment categories. In this study, regression analyses showed that alexithymia particularly mediated the relationship between symptom reporting and fearful attachment. Our results reinforce arguments to consider more clearly alexithymia and alexithymia subscales with an attachment view, closely linked to preoccupied and fearful categories, but not to dismissive category. This result echo studies, which explored he links between alexithymia and psychotic or neurodevelopmental troubles (which in our sample mostly composed dismissive category of attachment), and which reported that these disorders are not associated with alexithymia or only in specific sub-groups (Henry et al., 2010; Kinnaird et al., 2019).

#### 4.1.4. Attachment dimensions and categories with defence mechanisms

The distribution of defence mechanisms according to the axes of attachment in our study suggests a strong coherence between these two theoretical systems, which are described from different angles. However, there are very few studies in the literature to which our results can be compared, probably because of the important theoretical differences from which these concepts originate. By revealing correlations of neurotic and immature defence mechanisms with the anxious dimension of attachment, our results reinforces previous results, in which the effect of attachment style was mediated by defence mechanisms: 30% of the effect of fearful and preoccupied attachment styles on psychological distress in a sample of young healthy adults was mediated by immature and neurotic defences. Attachment style and defence mechanisms accounted for nearly 25% of the variance in psychological distress (Ciocca et al., 2020). Moreover, our study is in line with studies on BPD, in which described defence mechanisms included devaluation, passive aggression, omnipotence, primitive idealization, projective identification, retroactive cancelation, projection and cleavage. These immature mechanisms (Kernberg, 1975) had been linked to the BPD interpersonal skills impairment, including a strong dependency (Perry and Cooper, 1986; Presniak et al., 2010), the difficulty to express their needs to others, the tendency to project one’s internal states onto others, and the cleavage of the Ego with what it implies of devaluation of the self. Our results suggest a strong interaction between attachment dimensions or categories and defence mechanisms, and call for further investigation of these interactions.

### 4.2. Synthesis

In sum, our results suggest the existence of four emotional profiles. 1. In secure attachment, subjects are connected to their emotions, and to those of others. They have low levels of defence mechanisms, and may regulate emotions with the help of their internal resources and those of others: it is a *self-and-other oriented emotion perception*. 2. In preoccupied attachment, subjects are disconnected from their emotions (alexithymic) but strongly connected to the emotions of others: it is an *other-oriented emotion perception*. It suggests that the high level of neurotic defence mechanisms in these patients allows an adaptation to insecurity, by affiliation to others’ emotions and expectations, with a relative maintenance of reality testing. 3. In dismissing attachment, subjects are disconnected from others’ emotions and expectations, but have maintained a connection to their own emotions: it is a *self-oriented emotion perception.* The place of mature defence mechanisms in their functioning argues for autonomic attitudes, but, in this insecure context, it may be at the cost of a loss of reality resting. 4. In fearful attachment, patients are disconnected from their emotions, as well as from the emotions of others, with a high level of immature defence mechanisms. These patients have very few resources to cope with stressful situations: they are *disconnected from emotions*.

Our results echo theory and practical evidence that have described psychopathology in a transdiagnostic manner (Norton and Paulus, 2016; Norton and Roberge, 2017; Pearl and Norton, 2017). After having analyzed psychiatric disorders in a categorical way, some authors have, in line with Barlow et al. (2004), indeed defended the interest of a unified perspective of emotional disorders. Our observations pursue this reflection by highlighting the extent to which attachment could play an underlying but fundamental role.

### 4.3. Emotion regulation in adolescents

The adolescent status of patients in this study questions the effect of cerebral maturity in emotion regulation at an age where emotional consciousness in only emergent (Lerner and Steinberg, 2004). The large time lag between amygdala maturity and prefrontal cortex maturity, which characterizes neuronal development in healthy adolescents (Giedd et al., 1999; Sowell et al., 1999), has sometimes been suggested to play a role in adolescent difficulties in emotional information processing. In particular, parietal and frontal lobe areas have been shown to handle experiences of dynamic emotional stimuli, while temporal and limbic-related cortices process information about static emotional stimuli (Adolphs et al., 2003), raising the possibility that adolescents may be less sensitive to some stimuli than adults. Our results reinforce this hypothesis, in describing a positive correlation between age and sensitivity. Finally, patients’ sex ratio in this study has shown a possible influence on empathy and defence mechanisms, which is in agreement with the literature on gender and empathy or emotion recognition (McClure, 2000). Most published studies on emotion recognition have only included women and further studies should analyze emotional recognition in male adolescents, who are supposed to have more difficulties in facial expression processing in general as well as in BPD (McClure, 2000). Finally, the difference we observed in defence mechanisms scores between young men and women should be explored further, but echoes the study from Paris et al. (1996), in which men with BPD had comparable defence profiles to women with BPD (even if they did not have a lower adaptive profile score).

### 4.4. Limits and perspectives

The current study has certain limitations that must be considered. The sample size is limited to analyze the existence of subgroups. Some results failed to reach significance, probably due to this limit. However, descriptive data among attachment styles were interesting to observe independently of analyses, and were consistent with a number of data in the literature. Moreover, correlations analyses on the entire group were in line with data described in this four-classes analysis. Attachment categories were also measured using only a brief self-report questionnaire. Thus, participants’ responses reflected subjective evaluations rather than actual attachment strategies. Nevertheless, subjective representation is considered to be relevant to understanding the processes at work in psychopathology (Cassidy, 1994). Another limitation of the present study relates to the diagnosis assessment, which was not done after a structured interview, and is thus less reliable. Diagnostic instruments were not used for feasibility reasons (instability of patients). This prevented us from making more advanced comparisons between psychiatric diagnoses. Nevertheless, only one psychiatrist assessed the whole sample, according to DSM-5 criteria during their hospitalization, thus eliminating inter-rater discrepancy. Moreover, this psychiatrist is used to structured diagnostic interviews. The third main limitation was that most of our subjects were medicated, raising the question of the effect of medication on emotion perception. It is unclear whether psychotropic drugs can influence emotion identification, as the results of the few studies that looked at the impact of medication on emotion perception are very heterogeneous. Most of them however found no differences between medicated and unmedicated groups in adolescents and adults (Lynch et al., 2006; Dickstein et al., 2007; Guyer et al., 2007; Schenkel et al., 2007; Brotman et al., 2008a,b; Jänsch et al., 2009; Kalmar et al., 2009). In the present study, no link was found between the number of psychotropic treatments and emotion perception or defence mechanisms. It suggests that a potential medication effect on emotion perception would be secondary to attachment modalities. The convergence between FER skills and empathy, which have been measured in different ways – experimental and self-report questionnaire– (explicit knowledge is less easily influenced by medication than attention/perception modalities) argues for effects of psychopathology independently of medication. Finally, patients with heterogeneous medical diagnoses constitute this sample. Multiple data in the literature have pointed to the limitation of exploring emotional regulation within a diagnosis. Indeed, their results often showed heterogeneity of functioning within patients in one diagnosis, and the sample size (usually about 20–30 patients) did not allow the effect of comorbidities to be measured nor to conclude to a diagnostic specificity (Minzenberg et al., 2006; Domes et al., 2008; Kerr-Gaffney et al., 2019; McMahon et al., 2019). Thus, we voluntarily chose a transnosographic study for this reason, but recognize the inherent limitations of this choice in terms of the heterogeneity of the clinical sample. Despite these limitations, this study is, to our knowledge, the first to explore emotional perception and attachment styles in such a large sample of severe psychic troubles in adolescence in a transnosographic perspective. Patients in this sample are representative of typical adolescents hospitalized in a psychiatric department, with high severity and much comorbidity. These findings further our understanding of the differences in emotion perception in clinical conditions, including static and morphing stimuli, and constitute a basis of comparison for future research at this key period of development.

## Data availability statement

The datasets presented in this study can be found in online repositories. The names of the repository/repositories and accession number(s) can be found at: https://doi.org/10.6084/m9.figshare.19292051.

## Ethics statement

The studies involving human participants were reviewed and approved by Ethics Committee of Necker Hospital, Paris, France (CPP-IDFII-2013-09-10). Written informed consent to participate in this study was provided by the participants’ legal guardian/next of kin.

## Author contributions

MR and MC performed the conceptualization. MR and VP performed the methodology. VP, LS, LB, and MR were performed material preparation, investigation, and data collection. MR and VP performed the formal analysis. MR performed the original draft preparation, and LS, JB, CL, and MC reviewed it. MC performed the supervision. MR wrote the manuscript and all co-authors approved it. All authors contributed to the study conception and design.

## Conflict of interest

The authors declare that the research was conducted in the absence of any commercial or financial relationships that could be construed as a potential conflict of interest.

## Publisher’s note

All claims expressed in this article are solely those of the authors and do not necessarily represent those of their affiliated organizations, or those of the publisher, the editors and the reviewers. Any product that may be evaluated in this article, or claim that may be made by its manufacturer, is not guaranteed or endorsed by the publisher.
